# A Case of Double Vagina and Cervix Uteri

**Published:** 1879-11

**Authors:** Wm. R. D. Blackwood


### A CASE OF DOUBLE VAGINA AND CERVIX UTERI.

By WM. R. D. BLACKWOOD, M.D.

On the 10th of October, 1878, I was consulted by Miss ——, aged 22, with the appearance of robust health, for relief from a distressing headache, frontal in situation, moderate in intensity in the early morning, with increasing severity towards evening, at which time it usually disappeared, although occasionally the malady would continue far into the night, with the effect of banishing sleep till nearly the brake of day. In addition, she endured pain of varying force, indefinitely ascribed to the left hypogastric region. I say indefinitely, because it rarely, for an hour at a time, was constant at any one definite point. After being under my charge for some weeks, it became apparent that the difficulty was most probably located in or near the left ovary, which was more than usually tender upon pressure, and more than ordinarily movable through manipulation, although, left to itself, no tendency to downward displacement existed, as might under the circumstances have been expected.

Upon commencing a digital vaginal investigation, the first noticeable point was the unusually firm hymen, with, as proved by ocular demonstration, two openings therein. By careful, slowly-made, and gentle effort the left opening was dilated sufficiently to permit the finger to reach the cervix uteri, which at once gave evidence of abnormal developement, being sharply latero-flexed. Conjoined manipulation by the rectum proved that no displacement of the body of the uterus was present, and this led to a more careful procedure, the result of which developed the fact that each of the openings of the hymen entered vaginæ of their own. So far the case was one of double vagina. The time and pain prevented further investigation until next day, when a thorough dilatation of both cavities was made and a speculum examination begun. Each vagina was such as is found in the virgin, moderately capacious, and presenting no abnormal features. The cervix on the right side presented at an angle of about ten degrees, bending towards the right, and that of the left side made an angle of probably twenty degrees, pointing towards the left. Each cervix was normal in shape, size, and general characteristics, both canals patulous to the sound, but only so far as the os internum, beyond which, passage at this time was impossible with any justifiable force. Conjoined manipulation by both the rectal and abdominal parietes showed the uterus to be single, with double cervix. The body of the uterus was of the usual size, and was freely movable and not sensitive to pressure. No uterine catarrh existed; the organ was evidently sound. At the third examination, which occurred after an interval of three days, the right cervical canal was dilated instrumentally, and the sound passed to the fundus, the length being two and a half inches. The other cervix gave more trouble, but through careful and gentle manipulation the sound entered to a distance of two and three-quarter inches, the extra distance being clearly attributable to the lengthening of the cervix. There was a palpable difference in the feeling of the two sides of the cavity of the uterus after passing the internal os, a distinct elasticity or softness being experienced in the central line, the reason for which became apparent on introducing a sound into each cavity simultaneously. The uterus was divided into two cavities by a vertical septum, which is undoubtedly complete, no manœuvre allowing the sounds to touch each other through any discoverable opening. The case was thus shown to be one of *uterus septus*, the septum being prolonged to the ostium vaginæ. All other organs were apparently normal in position and action. It was determined to divide the vaginal septum. To obviate troublesome hemorrhage, the elastic ligature was resorted to. About one inch of tissue was included in the loop, which cut its way out in four days without much discomfort. The next time two inches were similarly treated, and the section was made as before in four days, the ligature requiring tightening on the second day. A third application severed another inch, leaving about one inch remaining between the right and left cervix. The idea of severing the intra-uterine septum also was entertained, but because of the thickness of the triangular mass of tissue between the two uteri, the operation was postponed, and nothing in that direction has yet been attempted.

In the general examination of the case I had elicited the fact that the pain which accompanied menstruation and was referred to the left side was intensified at every second menstrual period, and, although in every other respect no discomfort was experienced, there seemed here to be an implication of the left ovary; in fact, it was from this idea that the physical examination during the menstrual flow was suggested. The period in progress was the one in which the pain was not increased during the flow. The first point which interested me was, that the discomfort of the left side was less than it had been before menstruation set in, and that pressure was better borne, yet on the right side (over the ovary) firm pressure produced uneasiness to a greater degree than before the period. The thought occurred to me that possibly the congestion incident to ovulation might be confined to one only of the ovaries, and if such was the case, what effect, if any, would this have in the peculiar condition of the uterus? On very careful speculum examination the fact became apparent that the flow was entirely confined to the right side of the uterus. That every possible source of error should be excluded, I prolonged the examination for half an hour, after which time other engagements prevented further observation till the next day. On the succeeding afternoon I repeated the investigation, with precisely the same result, and, as the flow was much less abundant, the difficulty of keeping the parts clean was not so great as before. The small portion of the vaginal septum remaining permitted the plugging of the left Douglas cul-de-sac readily, and after retaining a tampon of absorbent cotton therein for half an hour, not a particle of blood had been extruded from the uterine cavity of that side. Similar results were obtained on the next day, the last of that period. For a day or two the lessened pain in the left side continued; it then recurred, however, and persisted until the next term ensued, when the trouble was intensified, as was habitual. Nothing of a surgical nature had been done in the interval, the only attention having been for the relief of the headache, which persisted, however, in spite of all that was done. On the appearance of the catamenia the specular examination was renewed. The flow was now from the left cervix alone. As before, care was had to exclude possible error, and during the three days ensuing the only difference observable was that the amount of discharge was less than had been observed from the right side, and some little pain was present during the first twenty-four hours. The present month has been the thirteenth since my inquiries began, and in these thirteen periods the regular alternation has been maintained, with a single exception—that in August, when the flow should have been right-sided, it was from the left, as in the month before.

*Remarks.*—The physiology of menstruation is not by any means thoroughly understood. Wide differences of opinion are held by eminent authorities on this subject, and it will doubtless require much study and research to unravel the hidden secrets of this exceedingly interesting branch of medical learning, and anything bearing upon the subject is therefore of right the property of the profession at large. The difficulty in accounting for congestion on one side only of a uterus divided simply by a septum of probably a quarter of an inch in thickness is hard to meet, and no explanation is attempted. That the excitation of the body of the uterus in this instance *is* thus confined is beyond question in my mind, for I can not conceive of the hyperæmia existing in each lateral half without the outpour of blood from each section. What the arterial and venous distribution is can not, of course, be here determined, but it must certainly be abnormal, or the septum must be of such a nature as to shut off communication between the sides by extending through the tissue of the organ to the peritoneal investment. Were the substance of the uterus bisected by a cartilaginous diaphragm, the enigma would at once be solved. If marriage in the case ensues (and I hope it will), and pregnancy occurs, the result will be very interesting; and it was largely from this line of probability that the portion of vaginal septum was allowed to remain, the idea suggesting itself that double pregnancy might take place at different periods. For a long time the headache and abdominal pain were apparently beyond relief; in fact, the latter was notably aggravated by the excitement kept up by frequently repeated examinations. Finally, however, electricity achieved an almost complete victory, and, from having to undergo daily treatment, the patient now often is for a fortnight free from trouble. It is probable that complete immunity will never be attained, owing to the physical peculiarities involved; but at any rate she is comparatively free from what had long been an inseparable companion —*Philadelphia Medical Times.*

